# Supplementary material for: Demonstration of CMOS-compatible memristor-based electrochemical biosensor transducer with threshold-sensing functionality
Source: Nat Commun. 2025 Dec 3;16:10851. doi: 10.1038/s41467-025-66372-w (PMC12675689; doi:10.1038/s41467-025-66372-w)
Supplement: Supplementary file 1 — Supplementary Information [file 41467_2025_66372_MOESM1_ESM.pdf]

Supplementary Information

Demonstration of CMOS-compatible Memristor-based Electrochemical Biosensor Transducer with Threshold-sensing Functionality

Young-Joon Kim[1,2‡], Youna Kwon[3‡], Youngwoo Yoo[1,2‡], Kandaswamy Theyagarajan[1,2], Sairaman Saikrithika[1,2], Aryeong Lee[4], Nam Ho Bae[3], Won-Chul Lee[3,5], Gapseop Sim[3,5], Younghyun Lee[6], See-On Park[7], Hyijae Cho[3,5], Min-Ho Kang[3], Youngjoo Kim[3], Yumin Park[3], Kyoung G. Lee[3], Choul-Young Kim[5], Hyoungho Ko[5], Woo-Suk Sul[3], Seok Jae Lee[3], Jae-Hyuk Ahn[5], Shinhyun Choi[7]*, Kyung Min Kim[6]*, and Jongwon Lee[8]**

[1]Department of Electronic Engineering, Gachon University, Seongnam 13120, Republic of Korea

[2]Department of Semiconductor Engineering, Gachon University, Seongnam 13120, Republic of Korea

[3]Division of Nano Convergence Technology Development, National Nanofab Center (NNFC), 291 Daehak-ro, Yuseong-gu, Daejeon, 34141, Republic of Korea

[4]Department of Materials Science and Engineering, Chungnam National University (CNU), 99 Daehak-ro, Yuseong-gu, Daejeon, 34134, Republic of Korea

[5]Department of Electronics Engineering, Chungnam National University (CNU), 99 Daehak-ro, Yuseong-gu, Daejeon, 34134, Republic of Korea

[6]Department of Materials Science and Engineering, Korea Advanced Institute of Science and Technology (KAIST), 291 Daehak-ro, Yuseong-gu, Daejeon, 34141, Republic of Korea

[7]School of Electrical Engineering, Korea Advanced Institute of Science and Technology (KAIST), 291 Daehak-ro, Yuseong-gu, Daejeon, 34141, Republic of Korea

[8]Department of Semiconductor Convergence, Chungnam National University (CNU), 99 Daehak-ro, Yuseong-gu, Daejeon, 34134, Republic of Korea

‡ These authors contributed equally to this work.

*** Correspondence and requests for materials should be addressed to Y.-J.Kim, S.Choi, K.M.Kim, and J.Lee.** (Email: Y.-J.Kim: youngkim@gachon.ac.kr, S.Choi: shinhyun@kaist.ac.kr, K.M.Kim: km.kim@kaist.ac.kr, J.Lee: jwlee80@cnu.ac.kr)

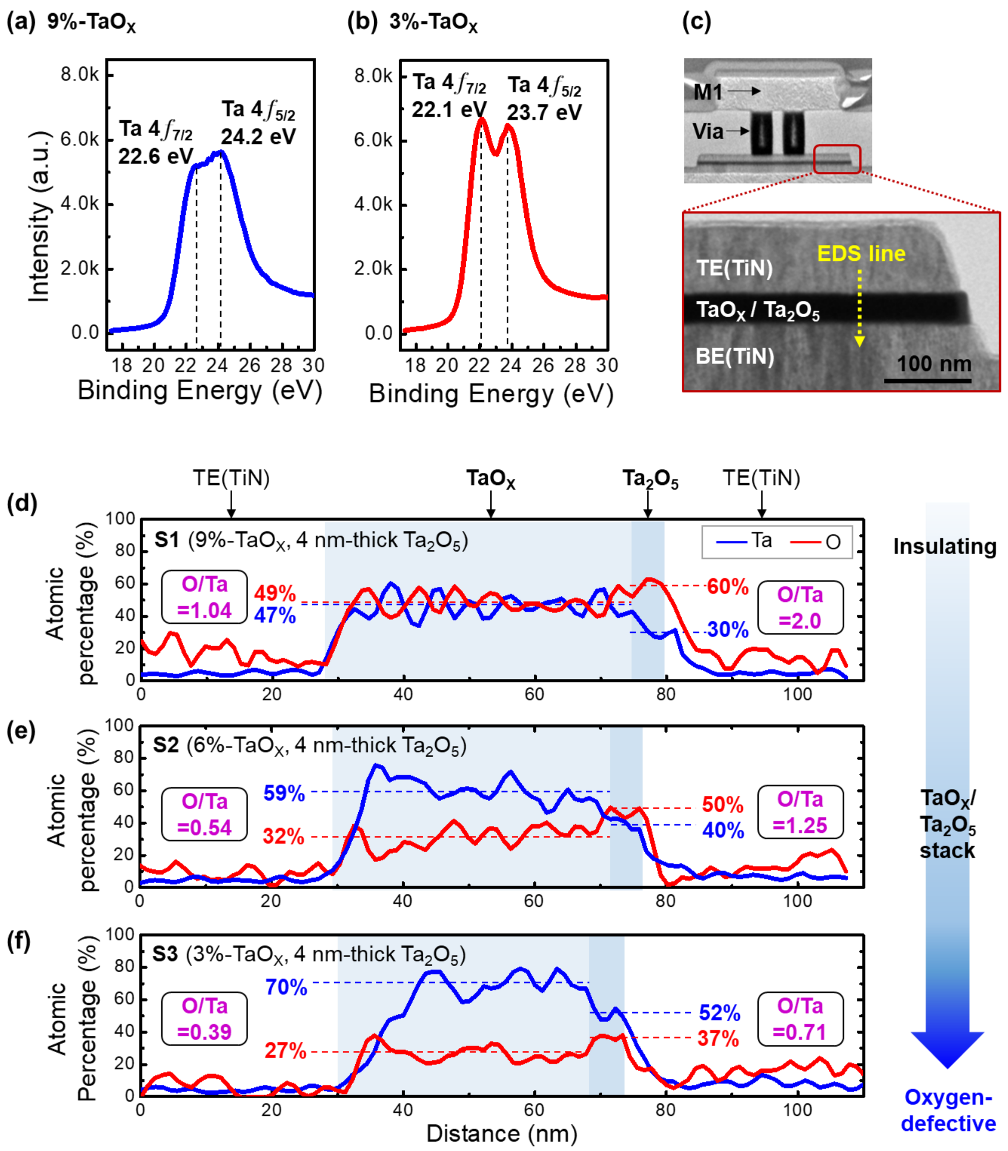

Supplementary Figure S1. The measured composition ratio of switching layer (SW) stack, TaO_X/Ta_2O_5 in the fabricated memristor species of S1 to S3. a and b. The spectra results of X-ray Photoelectron Spectroscopy (XPS) of the Ta 4f for single TaO_X layers with O_2/Ar gas ratios of 9% (9%-TaO_X) and 3% (3%-TaO_X). The peaks of binding energies of Ta 4f for 9%- and 3%-TaO_X layers were within the range of 22.1 and 24.2 eV, demonstrating that Ta-rich substoichiometric phases (TaO_X) were formed rather than stoichiometric tantalum oxide (Ta_2O_5) with a peak range of 26 to 29 eV for Ta 4f[1-2]. In addition, compared to the peaks obtained for 9%-TaO_X, the obtained peak of 3%-TaO_X is shifted by about -0.5 to -0.6 eV. This shift phenomenon of peak binding energies agrees well previously reported experimental results for the TaO_X layers[2]. **c.** TEM images of the fully fabricated memristor using standard CMOS back-end-of-line (BEOL) process. The composition ratio of each SW stack for three memristors of S1 to S3 was figured out, conducting EDS line-scan for the whole stack of $TiN/TaO_X/Ta_2O_5/TiN$. **d-f.** The EDS line-scan results showing atomic percentage profile versus distance for SW stacks with the reactive sputtered TaO_X layers with O_2/Ar gas ratio of 9% (S1), 6% (S2), and 3% (S3), respectively, and the ALD-deposited Ta_2O_5 layers. The Ta:O ratio in TaO_X for S1, S2, and S3 was measured to be 47:49, 59:32, and 70:27, corresponding to the mole fraction x of TaO_X of 1.04, 0.54, and 0.39, respectively. The Ta:O ratio in the ALD-deposited Ta_2O_5 layers was measured as 30:60, 40:50, and 52:37 for S1, S2, and S3, respectively, which indicated that the change in the O_2/Ar gas ratio in the TaO_X layer led to the change in the Ta:O ratio in the Ta_2O_5 layer. 3%-TaO_X made the Ta_2O_5 more oxygen-defective compared to 9%-TaO_X.

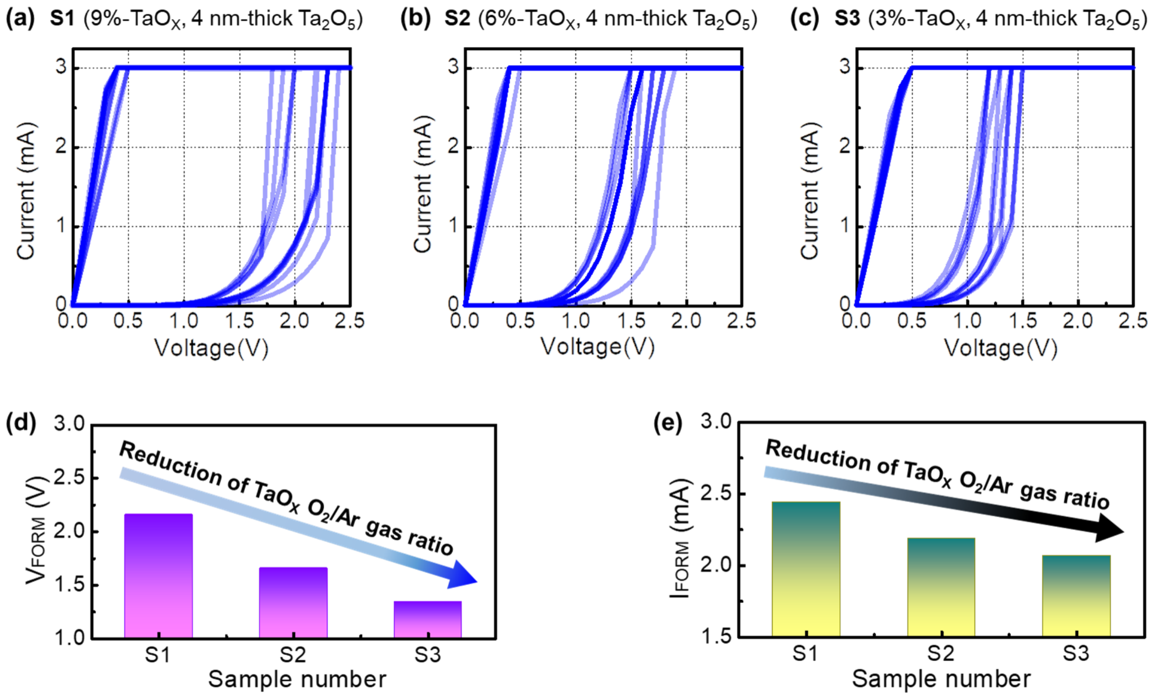

Supplementary Figure S2. Measured forming voltage and current characteristics of fabricated three TaO_X/Ta_2O_5 memristor species, S1 to S3, according to change of O_2/Ar gas mixture ratio of the TaO_X layer. a-c. Measured I-V curves of memristors with TaO_X layers with O_2/Ar gas mixture ratio of 9 % (S1), 6 % (S2), and 3% (S3). **d-e.** Measured average forming voltage and current values. The thickness of the Ta_2O_5 layer was kept the same as 4 nm for the three-types of memristors. For each type, ten devices were randomly selected on an 8-inch wafer. As the TaO_X O_2/Ar gas mixture ratio reduced from 9 % to 3 %, average forming voltage and current values of TaO_X/Ta_2O_5 memristors reduced.

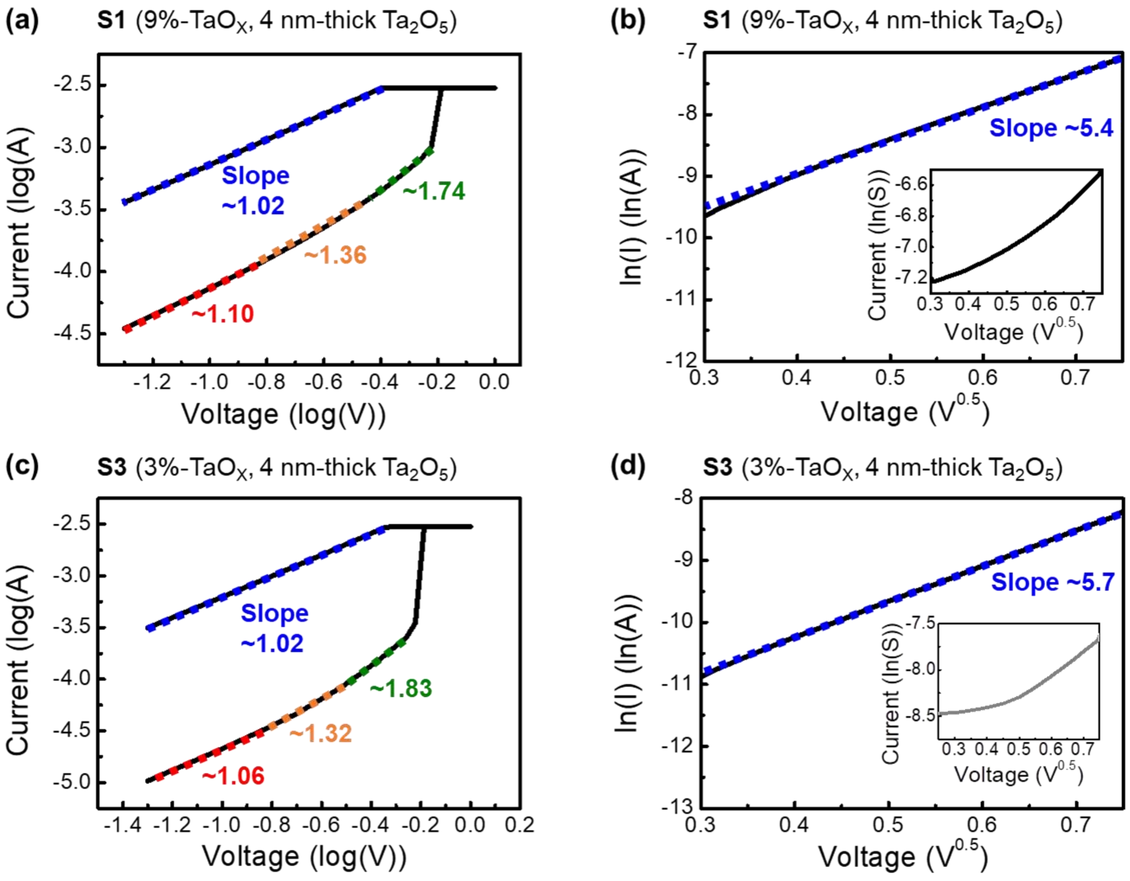

Supplementary Figure S3. Fitting results of the measured I-V curves to find out the conducting mechanism. a-b. Double-logarithmic and Schottky emission plots for the S1 species (with a TaO_X/Ta_2O_5 switching (SW) layer consisting of a 40 nm-thick TaO_X layer with O_2/Ar gas mixture ratio of 9 % and a 4 nm-thick Ta_2O_5 layer). The conduction mechanism of LRS in whole voltage range and of HRS in low voltage region was a metallic (ohmic) behavior due to the slope of 1 ~ 1.1. However, nonlinear double-logarithmic I-V characteristic appeared at high voltage region (corresponding to voltages of more than -0.8 log(V) or 0.15 V) for HRS. The experimental data in HRS at high voltage region fitted well with Schottky emission given by[3-4]

$$I \propto T^2 \exp\left(q\sqrt{((qV)/(4\pi\varepsilon_r\varepsilon_O d))}/kT\right) \quad [1]$$

where, I is the current, T is the absolute temperature, q is the electronic charge, V is the applied voltage, ε_r is the dynamic dielectric constant, ε_O is the permittivity of free space, d is the thickness of a SW, and k is Boltzmann's constant. Fig. S3b shows a fitting result for the Schottky emission. The I-V curve in HRS matches linearly well with a slope of 5.4 in high voltage ranges (from V = 0.15 to V = 0.55 V). The inset shows the Poole-Frenkel emission plot for HRS, which does not fit linearly in any voltage range. **c-d.** Double-logarithmic and Schottky emission plots for the S3 species (with a TaO_X/Ta_2O_5 SW layer including a 40 nm-thick TaO_X layer with O_2/Ar gas mixture ratio of 3 % and 4 nm-thick Ta_2O_5 layer). As shown in the Figures, the S3 device shows almost the same fitting tendency as the S2 device. It should be noted that all devices fabricated this time, including S1 and S3, have the same conducting mechanism (that is, metallic in LRS and Schottky emission in HRS), regardless of change of the thickness and O_2/Ar gas ratio of TaO_X layer.

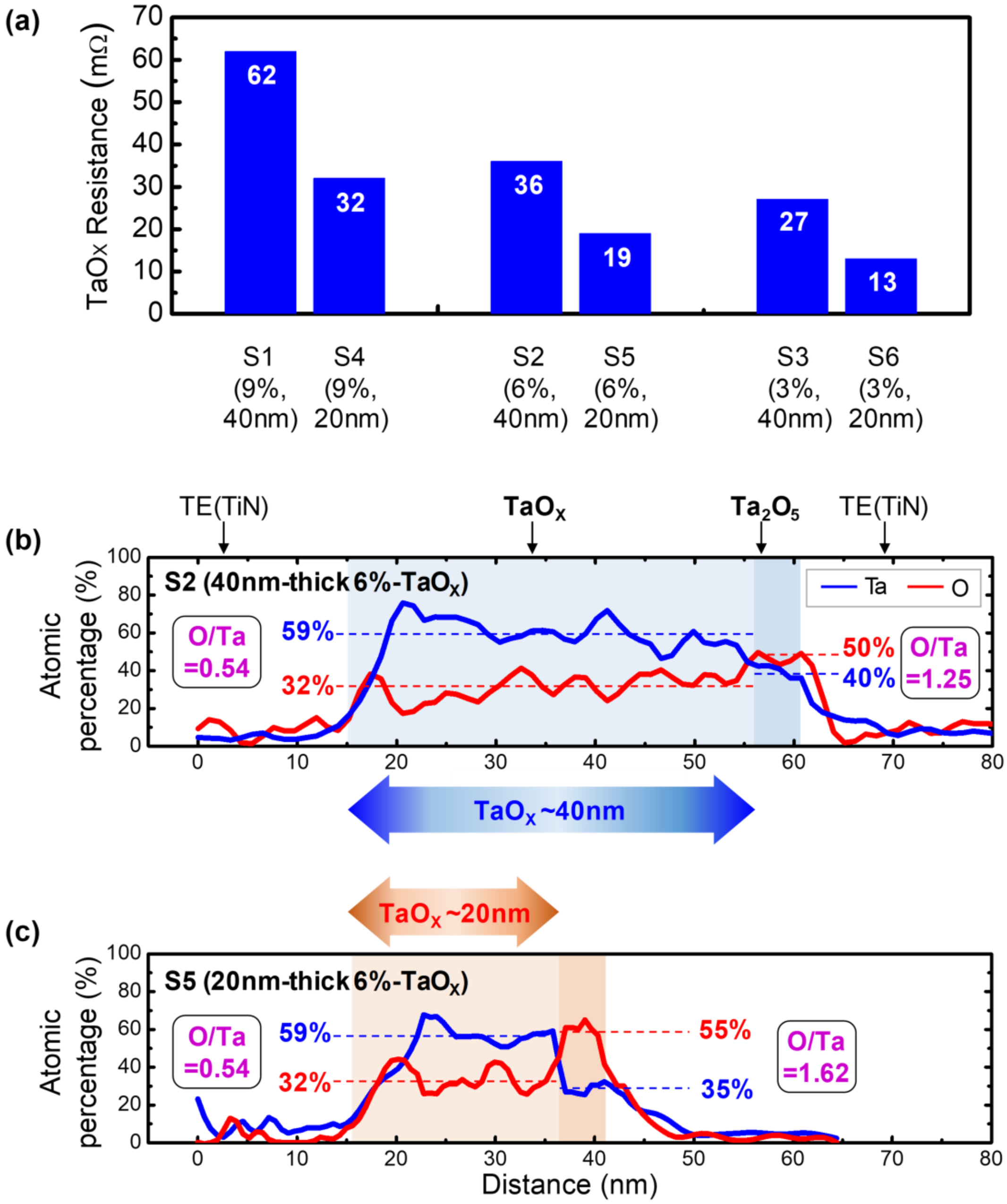

Supplementary Figure S4. The measured TaO_X resistance and composition ratio of the fabricated TaO_X/Ta_2O_5 memristors with different TaO_X thickness. a. The TaO_X resistance of single TaO_X layers with different process conditions in terms of O_2/Ar gas mixture ratio and layer thickness. The electrical resistance of 20 nm-thick TaO_X layers was approximately half of that of the 40 nm-thick TaO_X layers due to its thinner physical thickness. **b-c.** The EDS line-scan results showing atomic percentage profile versus distance of S3 and S6 species. S2 includes a reactive sputtered TaO_X layer with an O_2/Ar gas mixture ratio of 6% and a thickness of 40 nm (40 nm-thick 6%-TaO_X). S5 includes a 20 nm-thick 6%-TaO_X. The Ta_2O_5 layer in the S2 and S5 species had the same process condition with a thickness of 4 nm by ALD deposition. The O/Ta ratio in TaO_X for S2 and S5 was measured to be the same as 0.54.

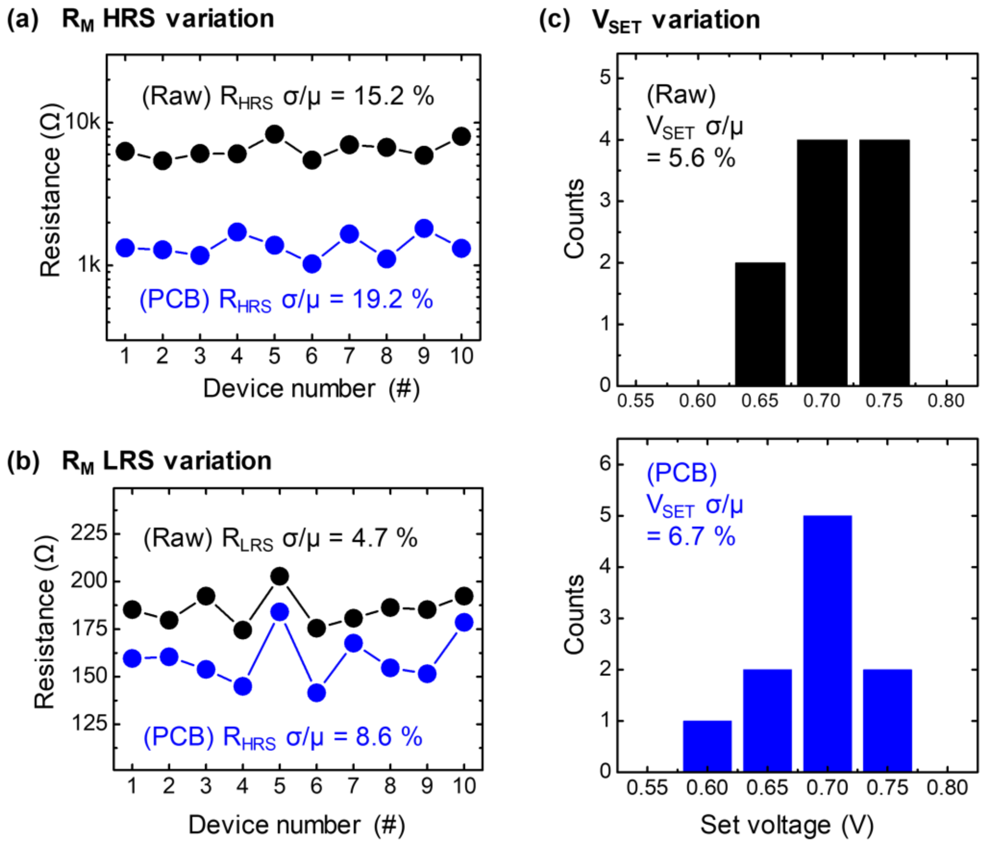

Figure S5. Measured variation characteristics of the fabricated S5 memristor devices. a. Device-to-device variation of the high-resistance state (R_{HRS}). **b.** Device-to-device variation of the low-resistance state (R_{LRS}). **c.** Device-to-device variation profile of the set voltage (V_{SET}). Both Raw memristors (from the S5 die) and PCB memristors (S5-die devices after PCB mounting) are shown together. The variation characteristics were extracted from 10 randomly selected devices. PCB memristors exhibited larger variation coefficients (R_{HRS}, R_{LRS}, and V_{SET} = 19.2, 8.6, and 6.7 %) than Raw memristors (15.2, 4.7, and 5.6 %), mainly due to extrinsic factors such as wire bonding and the absence of ES protection in the present biosensor configuration.

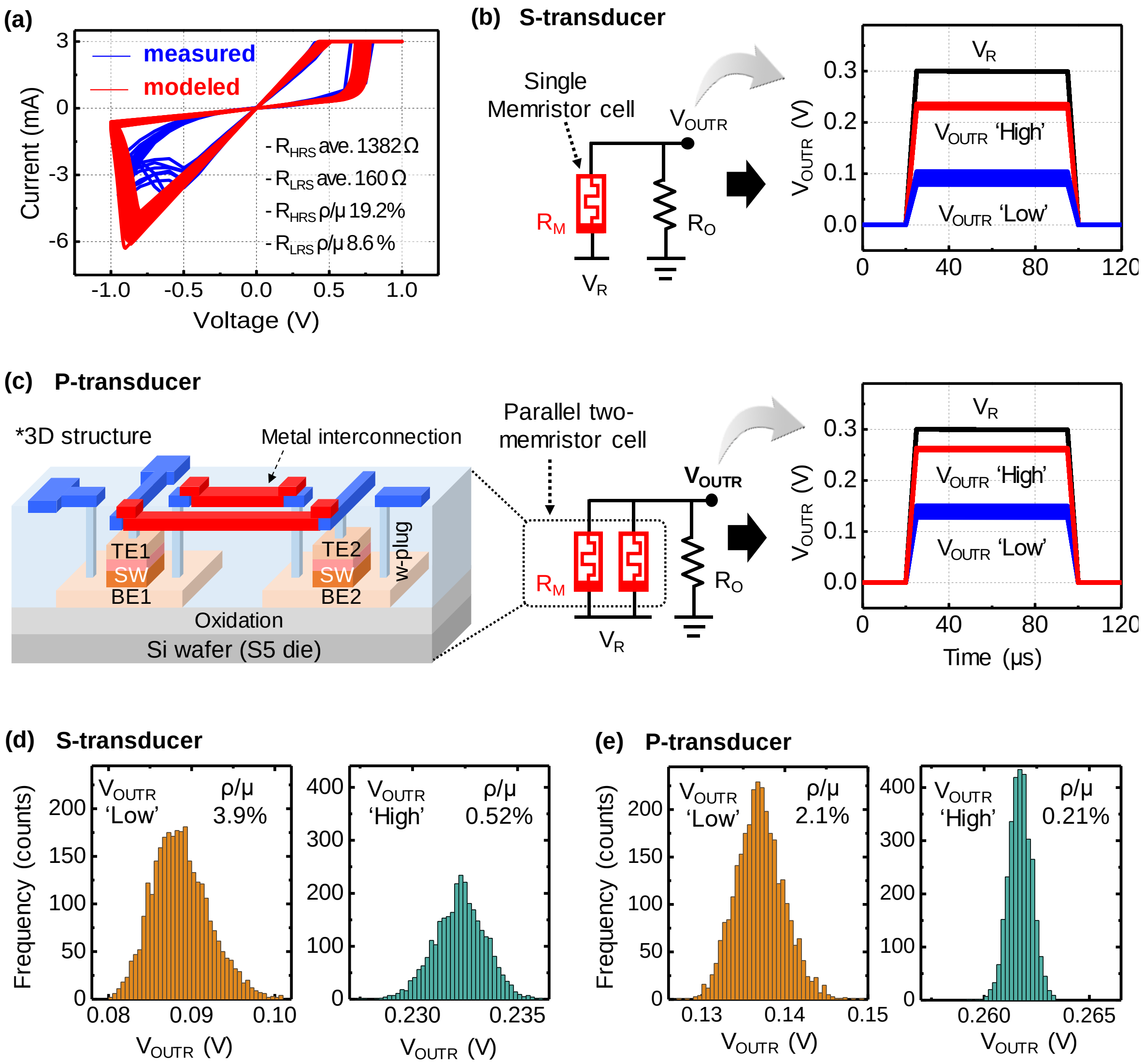

Supplementary Figure S6. The effect of the parallel two-memristor cell configuration on the biosensor transducer. a. I-V curve fitting results for measured and modeled memristors, showing good agreement in the positive voltage region, which directly contributes to the threshold operation of the transducer. **b-c.** Simulated transient characteristic for the output voltage in the reading stage (V_{OUTR}) of transducers based on a single-memristor cell (S-transducer) and a parallel two-memristor cell (P-transducer). The S-transducer and P-transducer exhibited voltage swings between the logic-high and logic-low levels of 140 mV and 120 mV, respectively, both of which satisfied the 100 mV criterion set in this work to verify the proper operation of the threshold-sensing function. The variation of V_{OUTR} at logic-high and logic-low levels decreased from 0.52% and 3.9% with the S-transducer to 0.21% and 2.1% with the P-transducer, respectively. This indicates that the P-transducer exhibited approximately 50% lower V_{OUTR} variation compared with S-transducer, which means a more stable transducer operation. The simulation in Figs. S6b-S6d was carried out using 3000 modeled memristor devices.

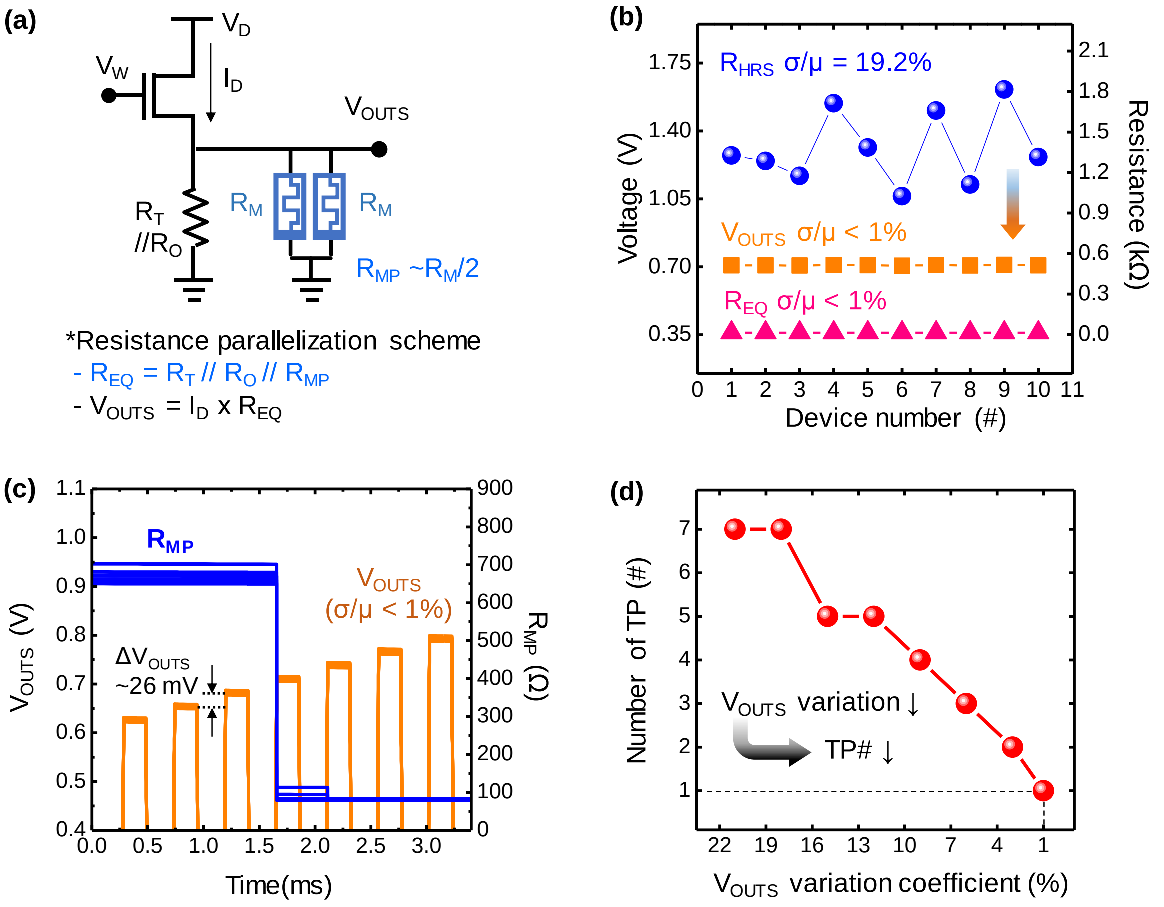

Supplementary Figure S7. The effect of the resistance parallelization scheme on the biosensor transducer. a. Circuit diagram of the biosensor transducer at the sensing stage. **b.** Simulated parallel resistance (R_{EQ}) and sensing-stage output voltage (V_{OUTS}) characteristics based on the measured R_{HRS} (HRS resistance of memristors) variation of 19.2%. The R_{EQ} variation coefficient was considerably reduced to less than 1%, resulting in a V_{OUTS} variation coefficient also below 1%. **c.** Simulated switching characteristics of the parallel memristor cell resistance (R_{MP}) under increasing V_{OUTS} with a variation coefficient of less than 1%. With V_{OUTS} increased in ~26 mV step, R_{MP} abruptly switched from ~700 Ω to ~80 Ω at about 700 mV, indicating the presence of only one threshold pH point (TP). **d.** Simulated number of TP as a function of the V_{OUTS} variation coefficient, confirming that V_{OUTS} variation of less than 1% ensures only one TP. The simulations in Figs. S7b-S7d were performed using 10 modeled memristor devices, and the set-voltage variation of the devices was neglected to examine the pure effect of V_{OUTS} variation.

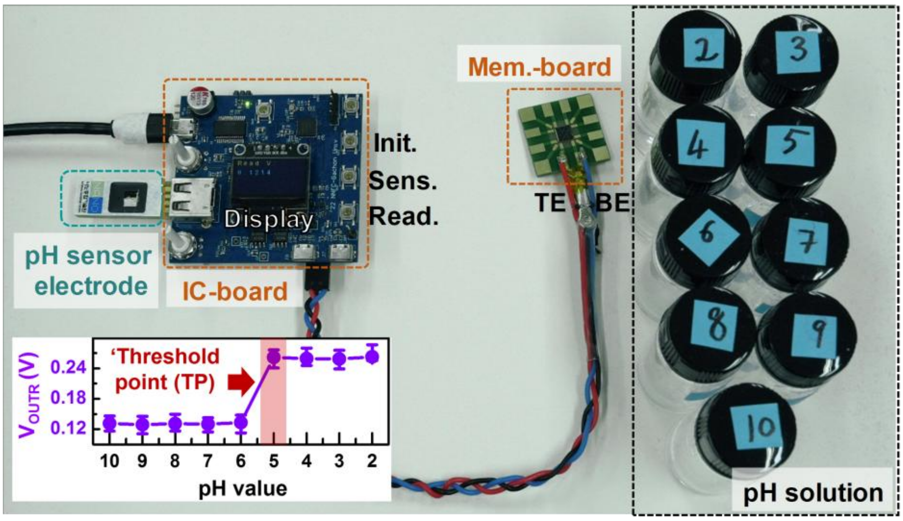

Supplementary Figure S8. Experimental setup of the demonstrated memristor-based fully integrated electrochemical biosensor system for pH sensing. The real-time operation of the system is shown in Supplementary Video 1, confirming successful threshold operation of pH 2-10 solutions, as presented in Fig. 6d with a threshold pH point at pH 5.

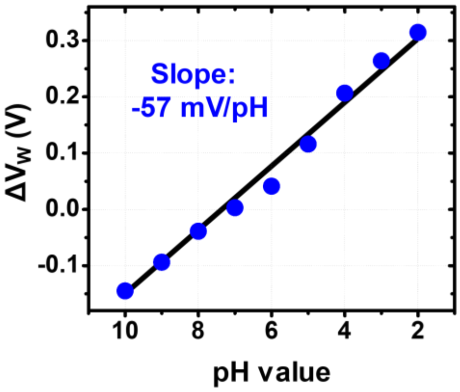

Supplementary Figure S9. The measured delta V_W (ΔV_W) versus pH characteristic. ΔV_W is a potential difference in V_W before and after dropping pH solutions with values between 2 and 10 onto a fabricated USB-type sensor electrode. As the pH decreased from 10 to 2 (i.e., the acidity increased), the ΔV_W increased linearly from -144.92 to 314.41 mV, exhibiting a close-to-linear behavior with a slope of approximately -57 mV/pH.

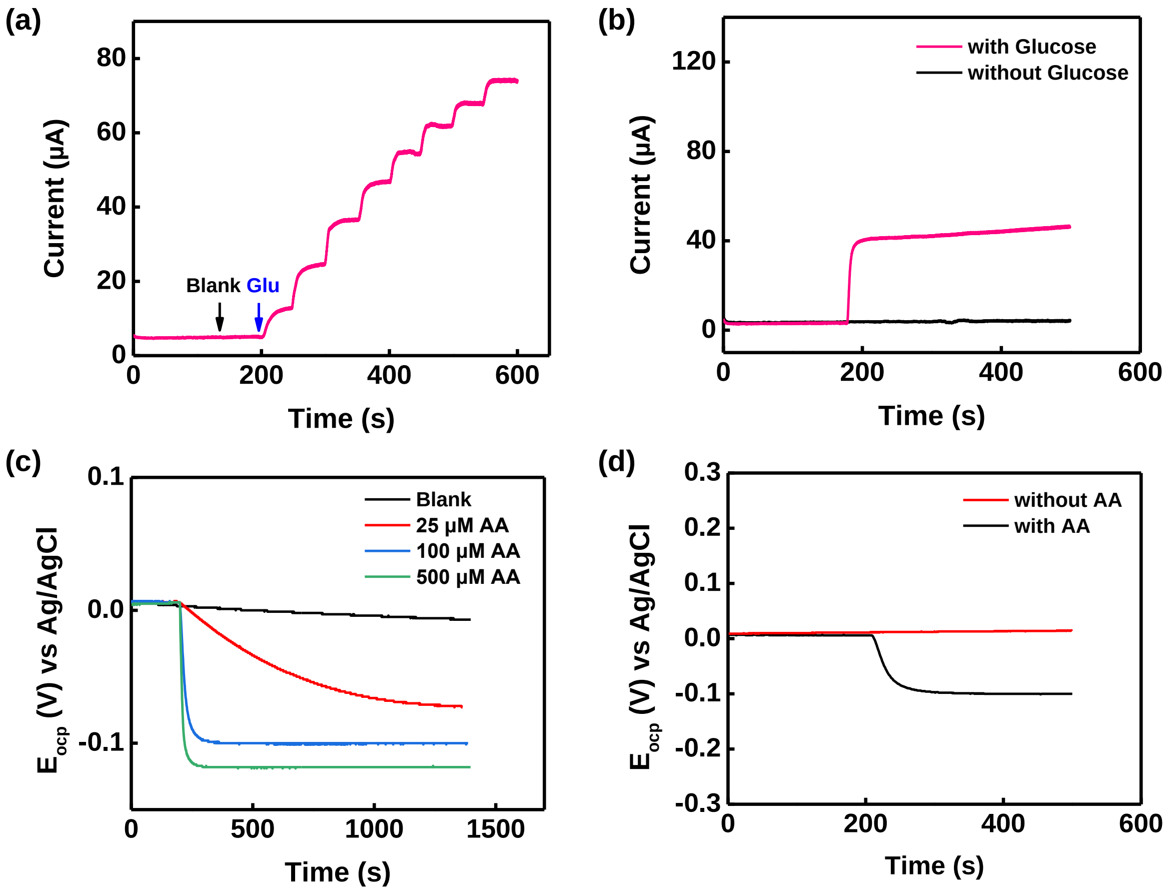

Supplementary Figure S10. Electrochemical biosensing responses of glucose and ascorbic acid. a. Amperometric i-t response of glucose sensor for the various concentrations of glucose (0.25 – 2.0 mM). For every addition of glucose, the sensor exhibited a rapid increase in catalytic current. **b.** The electrochemical stability of the developed sensor in the absence and presence of 1 mM glucose for 500 s, which showed excellent stability under dynamic conditions. Electrolyte: 0.1 M NaOH, Applied Potential: +0.4 V. **c.** The open circuit potential response of ascorbic acid (AA) sensor under dynamic conditions with different concentrations of AA (25 – 500 µM) in 0.1 M PBS. **d.** The electrochemical stability of the sensor was investigated in the absence and presence of AA, demonstrating excellent stability for 500s. These results confirm that the developed sensors exhibit excellent electrocatalytic activity and stability toward their respective target analytes. Further, the utility of the memristor was investigated towards the electrochemical biosensing of glucose and AA. When integrated with the memristor device, the sensor altered the device's resistance state upon reaching a predefined threshold. For demonstration purposes, we set thresholds of 1.25 mM for glucose and 100 µM for AA. In the case of glucose, the memristor retained its high resistance state (HRS) when the glucose concentration was below the threshold. Upon reaching the threshold concentration, the memristor switched from an HRS to a low resistance state (LRS) (Supplementary Video 2). Conversely, for AA, the memristor switched its HRS to LRS when the concentration of AA was below threshold, and upon reaching the threshold concentration, the memristor retained its HRS condition (Supplementary Video 3).

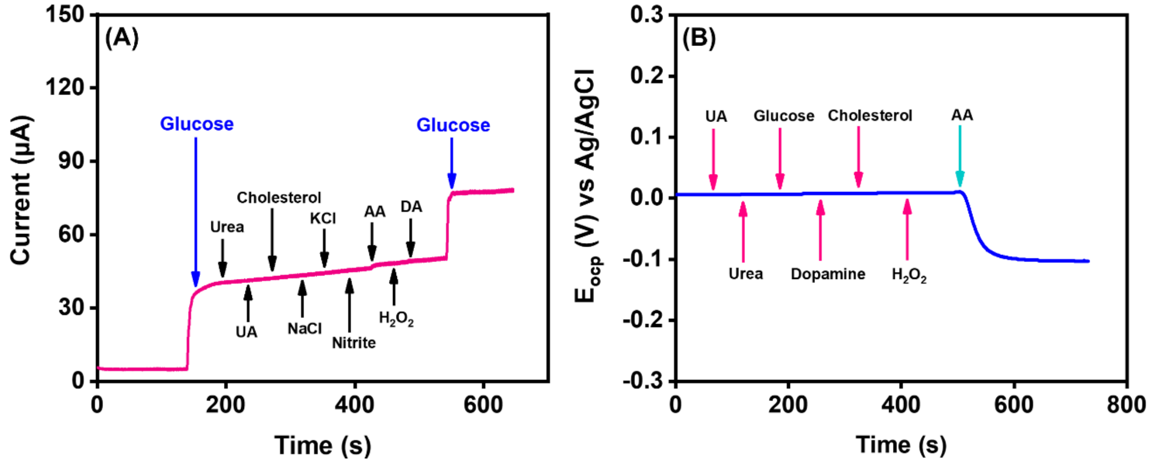

Supplementary Figure S11. Selectivity response of glucose and ascorbic acid biosensors. a. Amperometric i-t curve of the glucose sensor in the presence of various interferents, including urea, uric acid, cholesterol, NaCl, KCl, nitrite, ascorbic acid, hydrogen peroxide and dopamine in 0.1 M NaOH at +0.4 V. **b.** Open circuit potential curve obtained for the AA sensor in the presence of different interfering species such as uric acid, urea, glucose, dopamine, cholesterol and hydrogen peroxide in 0.1 M PBS. These results demonstrate that both sensors maintained high selectivity under the tested conditions.

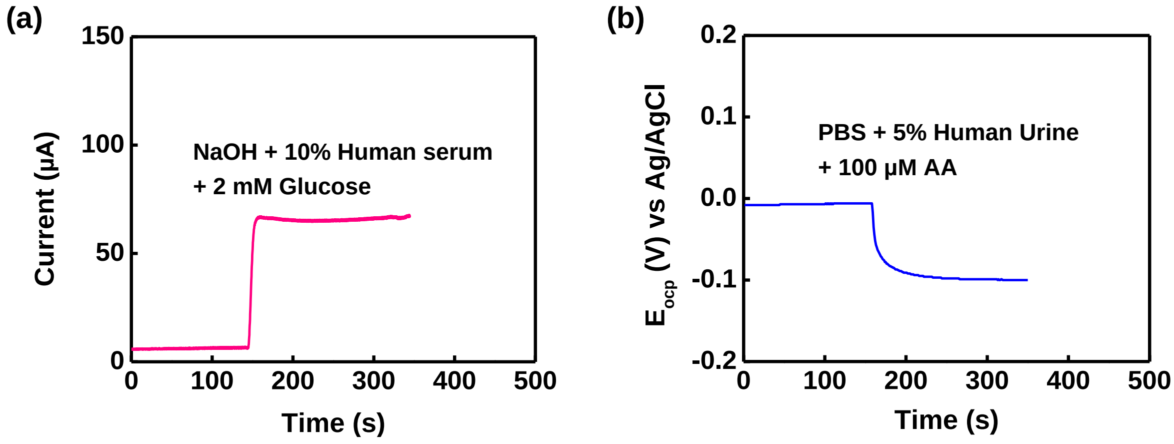

Supplementary Figure S12. Real-sample responses of glucose and ascorbic acid biosensors. (A) Amperometric i-t curve obtained for the glucose sensor for the addition of 2 mM glucose in 0.1 M NaOH containing 10% human serum at an applied potential of 0.4 V. (B) Open circuit potential response of AA sensor for the addition of 100 µM AA in 0.1 M PBS containing 5% human urine. The sensors exhibited stable performance in these complex biological matrices, demonstrating the suitability of the proposed memristor-based biosensor for detecting relevant biomolecules.

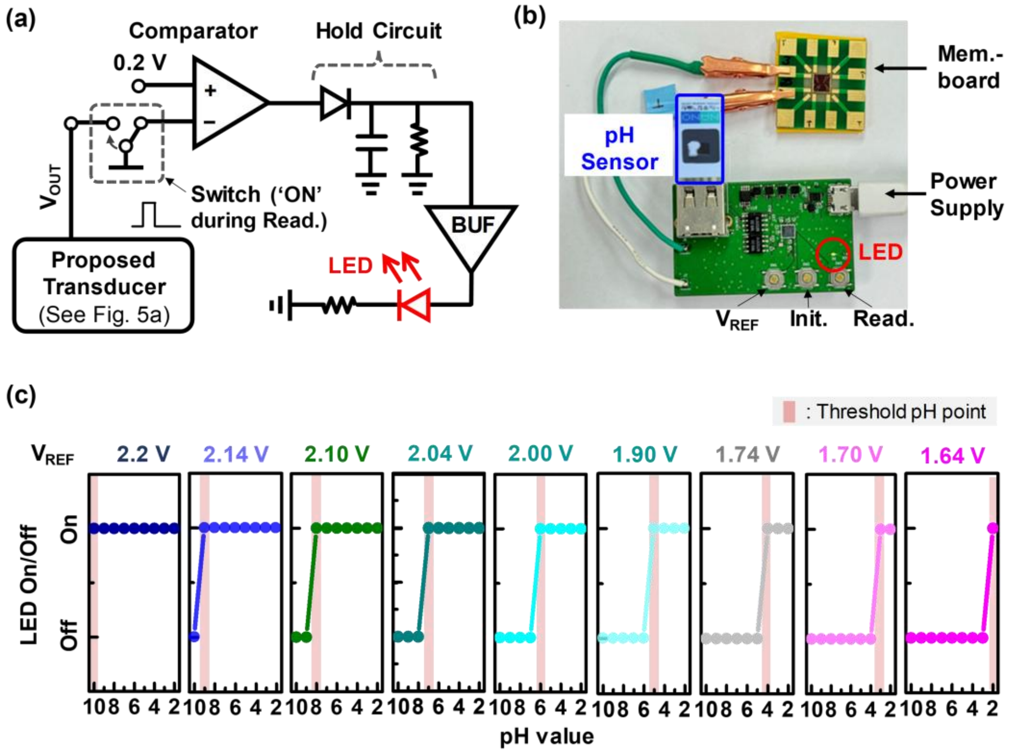

Supplementary Figure S13. Implementation of a compact version of the memristor-based electrochemical biosensor system with threshold-sensing functionality. a. A system diagram of the compact biosensor system. To implement the memristor-based biosensor more simply, the system employs a comparator and an LED instead of the ADC and the display shown in Fig. 5a. If the output voltage (V_{OUT}) exceeds a predefined threshold of the comparator of 0.2 V, the comparator outputs a high voltage. A hold circuit consisting of a diode, capacitor, and resistor extends the duration of the high voltage, allowing the LED to remain on even after the reading stage, which has a short duration of only 80 μs, is over. **b.** An image of an implemented prototype of the compact biosensor system. The LED lights up when the V_{OUT} exceeds the threshold of 0.2 V. **c.** pH measurement results of the compact biosensor system according to changes in V_{REF}, demonstrating that the threshold pH point is changed well in units of pH 1 by adjusting the V_{REF} value.

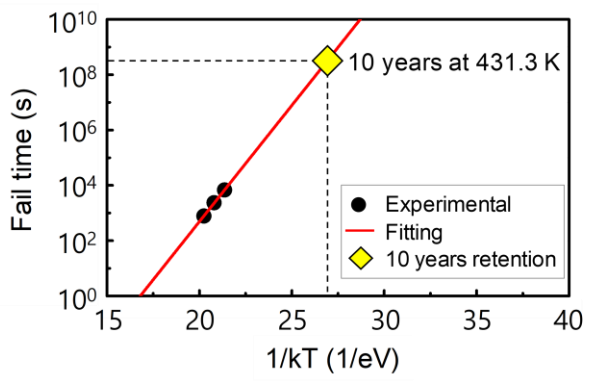

Supplementary Figure S14. Retention performance of the fabricated memristor, demonstrating reliable non-volatile behavior with an extrapolated retention time of 10 years at approximately 431.3 K. The retention was extracted by tracking the failure time at elevated temperatures (573, 558, and 543 K) and extrapolating the fitted data to lower temperatures.

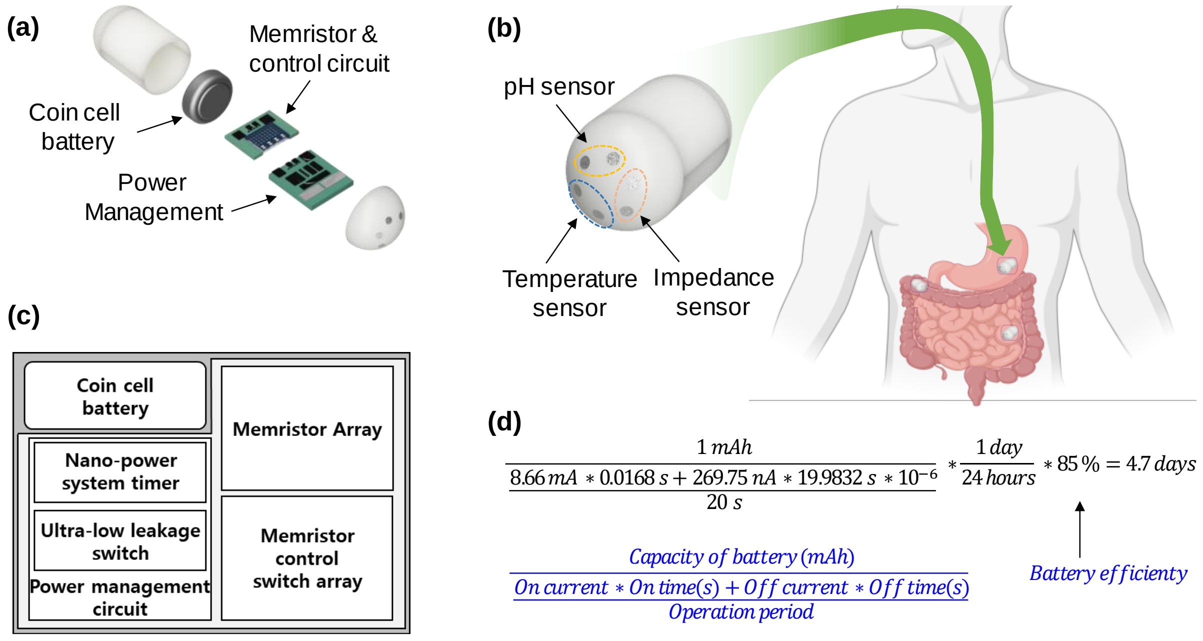

Supplementary Figure S15. Potential application and future outlook of the memristor-based electrochemical biosensor transducer with threshold-sensing functionality. a. Exploded view of memristor integrated ingestible sensor capsule. **b.** Schematic illustration of ingestible sensor capsule including multimodal sensors suitable for the physiological parameters within the gastrointestinal tract[18,19]. Figure partially created in BioRender. Kim, Y. (2025) https://BioRender.com/37pd34n. **c.** Block diagram of the sensor system. The ultra-low-leakage switch minimizes power loss during sleep mode. A nano-power system timer generates the clock signal for memristor operation and power switching, while a switch array manages the memristor array and its programming sequence. **d.** The estimated battery life equation and the estimated number of batteries when the measurement of the 20-second cycle is performed.

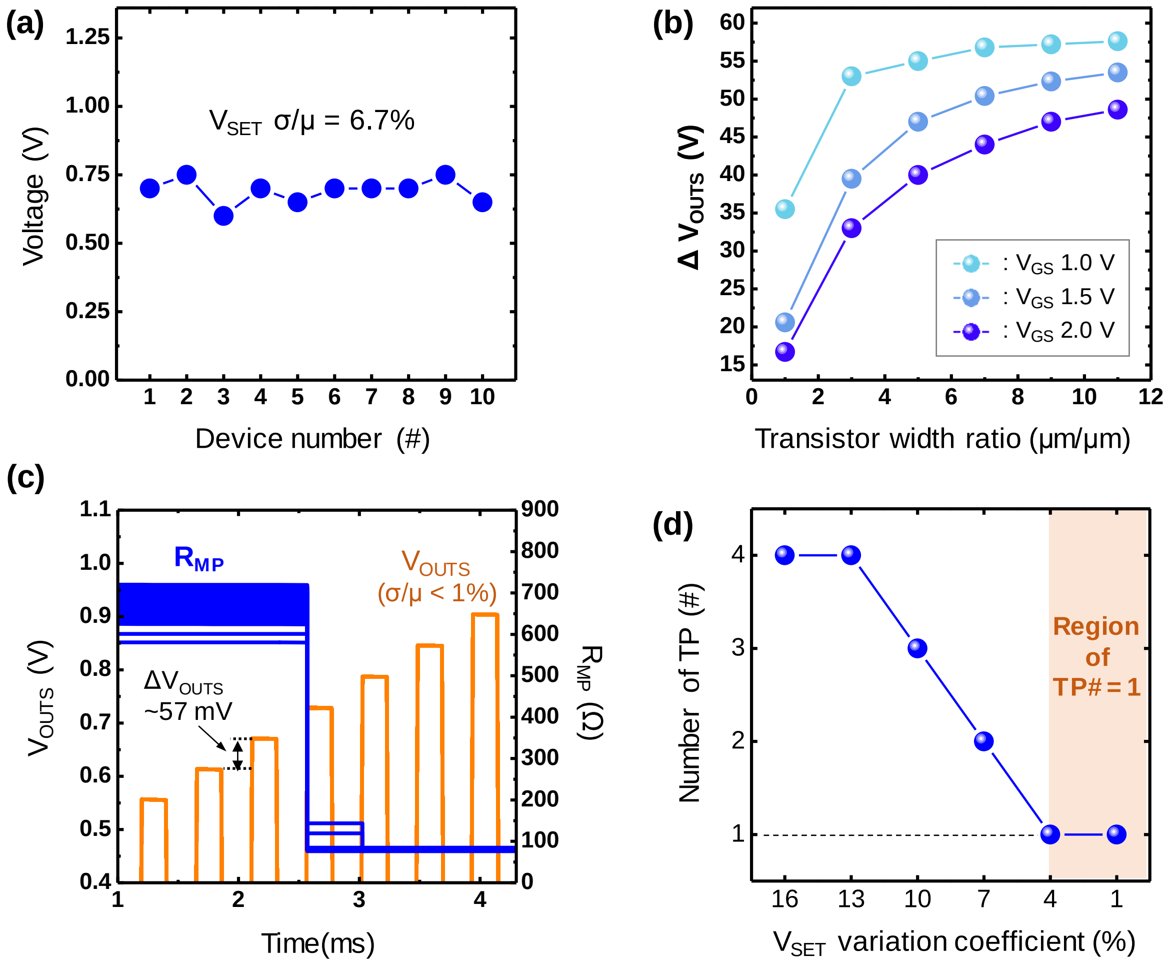

Supplementary Figure S16. Strategy for biosensor quality control against memristor set voltage variation. a. Measured set voltage (V_{SET}) variation for ten PCB-mounted memristor devices. The D2D variation coefficient of ten PCB-mounted memristor devices was measured to be 6.7% **b.** Optimization of transducer circuit design by adjusting the gate width ratio of the series-connected transistor pair and the gate bias (V_{GS}) of the lower transistor. The gate width ratio is defined as the geometrical gate width of the upper transistor divided by that of the lower transistor, as shown in Fig. 5a. An increase in the gate width ratio increased ΔV_{OUTS} from 26 mV to 57 mV. **c.** Simulated operation of the threshold-sensing (TS) biosensor transducer with three elements for effectively reducing V_{SET} variation: a memristor V_{SET} variation coefficient of less than 4%, a ΔV_{OUTS} exceeding 50 mV, and the use of only devices within the ±1σ range of the V_{SET} distribution, representing the effect of a system-level algorithm. Parallel memristor resistance (R_{MP}) switched from HRS to LRS at a single step of about 700 mV, indicating the presence of only one threshold pH point (TP). The detailed explanations regarding the three elements for mitigating V_{SET} variation are presented in Supplementary Note S1. **d.** Simulated number of TP of the biosensor transducer as a function of the memristor V_{SET} variation coefficient under the same conditions as in **c**, indicating that memristor V_{SET} variation coefficient below 4% enables the biosensor to achieve error-free threshold detection. The simulation in **c** and **d** was performed with 200 modelled memristor devices.

	Fully-integrated biosensor system	Simplified biosensor system
Used peripheral	GPIO, ADC, I^2C	GPIO
Method of sensing the memristor cell	ADC	Comparator
Method of user feedback	OLED display	LED
Tested pH range	2 - 10	2 - 10
Tested pH interval	1 pH	1 pH
The number of threshold pH point	1	1
Power consumption	17.95 mA	1.45 mA
Active size of IC board	60 × 60 mm^2	30 × 30 mm^2

Table S1. Experimental comparison between the fully-integrated and simplified versions of the biosensor systems. The simplified system achieved approximately a 4-fold reduction in IC board size and a 12.4-fold reduction in power consumption, relative to the fully-integrated system. Such a simplified system design is expected to be utilized for biosensor applications that require ultra-miniaturization and ultra-low power operation, such as ingestible sensor capsules.

Ref	Type of Device	CMOS compa-tibility	Device Reten-tion	Device On/off ratio	TS in trans. ducer	Appli-cation	Demo. func-tion	Demo. level	Sensor sensi-tivity	Sensor linea-rity
7	In-organic nano-wire	No	No	No	No	Electro-chemical bio-sensor	PSA	Device	N/A	N/A
8	In-organic nano-wire	No	No	No	No		DNA	Device	N/A	N/A
9	In-organic graphene transistor	No	No	No	No		Heart failure	Device	N/A	99.17 %
10	In-organic EG-FET	**Yes**	No	No	No		Influen za virus	Device	26.7 mV/ dec	N/A
11	In-organic memristor ($Pt/Al_2O_3/TiO_2/Pt$)	No	N/A	N/A	No		PSA	Device	N/A	N/A
12	In-organic memristor (IrO_x/GdO_x//W)	No	N/A	~1000	No		pH & Urea	Device	53.2 mV/ pH	98.98 %
13	In-organic memristor ($Pt/NbTiO_x/NbTi$)	No	N/A	1~5	No		pH	Device	N/A	98.0 %
14	In-organic ISFET	**Yes**	N/A	No	No		pH	Device	53.98 mV/ pH	N/A
15	Organic nanowire (PU/AuN Ps)	No	No	No	No		pH	System	58.9 mV/ pH	99.99 %
16	Organic memristor (Au/MSF P/Au	No	Short-term (2.7 h)	10	No	Neuro-morphic in-sensor comput. system (training & classi-fication)	Image recog-nition	**System**	-	-
17	Organic memristor (PEDOT :PSS)	No	Short-term (1 h)	6	No		Genetic disease	**System**	-	-
18	Organic mem-transistor (PEDOT :PSS)	No	Short-term (1 s)	No	No		gesture recog-nition	**System**	-	-
19	Organic transistor (Poly)	No	Short-term (1 s)	200	**Yes**	Neuro-morphic neuron	Neuro-nal beha-vior	Device	-	-
This Work	In-organic memristor ($TiN/TaO_x/Ta_2O_5$)	**Yes**	**Long-term** (≥10 y)	35	**Yes**	Electro-chemical bio-sensor	pH, Glu-cose, Ascor-bic acid	**System**	57 mV/ pH	98.1 %

Table S2. Comparison with previously reported semiconductor-based biosensors. Our biosensor represents, to the best of our knowledge, the first semiconductor device-based electrochemical biosensor that demonstrates threshold-sensing (TS) functionality in the transducer level together with complete system-level integration. Our memristor offers full CMOS compatibility, which is readily extendable to monolithic integration with SP blocks, facilitating the realization of miniaturized sensor systems. Moreover, our transducer is the only one that simultaneously exhibits intrinsic TS functionality and long-term non-volatility (Supplementary Fig. S14), which is valuable for miniaturized sensors with self-diagnosis capability.

Supplementary Note S1

Quality control measures to effectively mitigate V_{SET} variations as a future plan

This work was conducted as a proof-of-concept study for the proposed biosensor transducer, and therefore measures to address set voltage (V_{SET}) variation of the memristors were not included within the present scope. Effective control of V_{SET} variation is critical for ensuring reliable biosensor operation. To clarify our future directions, we have added a discussion describing three possible approaches for mitigating V_{SET} variation, as summarized below:

Approach 1) Application of filament-confinement memristors with low V_{SET} variation.

In this work, the D2D variation coefficient of ten PCB-mounted memristor devices was measured to be as low as 6.7% (see Supplementary Figures S5c and S16a), which we attribute to the use of a stable 180 nm CMOS BEOL process and the low Gibbs free-energy-based redox reactions of the Ta_2O_5/TaO_X switching layer. Recent studies have demonstrated remarkable progress, with memristors exhibiting V_{SET} variation coefficients as low as ~4%[20].

Approach 2) Optimization of transducer circuit design.

In this work, the measured ΔV_W was 57 mV/pH, which is comparable to previous results (see Figure 6d). However, this ΔV_W of 57 mV was reduced to a ΔV_{OUTS} of ~ 26 mV after passing through a series-connected transistor pair shown in Fig. 5a. For achieving high diagnostic accuracy of the biosensor, it is essential to secure a wide ΔV_{OUTS} that can encompass V_{SET} variation. In this work, the gate width ratio between the upper and lower transistors of the series-connected transistor pair was set to 1. By increasing this gate width ratio to more than 10, together with gate-bias optimization of the lower transistor, a ΔV_{OUTS} exceeding 50 mV was obtained in our transducer simulation (see Supplementary Figure S16b). This improvement is attributed to the enhanced trans-conductance (g_M) of the upper transistor due to its increased geometrical width, which represents a general device-level design technique to improve g_M.

Approach 3) System-level algorithm for mitigating memristor variability.

V_{SET} variation of memristor ReRAM devices generally follows a Gaussian distribution, and devices located in the distribution tail degrade the diagnostic accuracy of the biosensor. To address this, we apply a system-level algorithm that enables reliable biosensor operation by utilizing only devices with V_{SET} distribution confined within $\pm 1\sigma$. This confinement will be realized through "sensor trimming", which will compensate for differences in the average V_{SET} values among devices by adjusting the V_{REF} voltage (as already demonstrated in Figure 6e of the main manuscript). Intra-device V_{SET} variation will further be managed by a "repeated-measurement and averaging" algorithm, in which each measurement is repeated more than ten times, the results are averaged, and outliers with large deviations are discarded.

When the three approaches are applied, namely, confining the memristor V_{SET} variation to below 4%, widening ΔV_{OUTS} to greater than 50 mV, and applying a system-level algorithm that effectively utilizes only devices within $\pm 1\sigma$, our biosensor is expected to achieve error-free threshold detection within an interval of one pH unit (see Supplementary Figures S16c and S16d).

Supplementary References:

1. Hur, J.-H. and Lee, D., Universal Memory Characteristics and Degradation Features of ZrO_2-Based Bipolar Resistive Memory, *Adv. Electron. Mater.* **6**, 2000368 (2020).
2. Simpson, R., White, R. G., Watts, J. F., and Baker, M. A. XPS investigation of monatomic and cluster argon ion sputtering of tantalum pentoxide, *Appl. Surf. Sci.* **405**, 79-87 (2017).
3. Lin, C.-Y., Wang, S.-Y., Lee, D.-Y., and Tseng, T-Y., Electrical Properties and Fatigue Behaviors of ZrO2 Resistive Switching Thin Films, *J. Electrochem. Soc.* **154**, H615-H619 (2008).
4. Zhang, Y., Wu, H. Bai, Y., Chen, A., Yu, Z., Zhang, J., and Qian, H., Study of conduction and switching mechanisms in Al/AlOx/WOx/W resistive switching memory for multilevel applications, *Appl. Phys. Lett.* **102**, 233502 (2013).
5. Tzouvadaki, I., Lu, X., Micheli, G. D., Ingebrandt, S., and Carrara, S., Nano-fabricated memristive biosensors for biomedical applications with liquid and dried samples, *2016 38th Annual International Conference of the IEEE Engineering in Medicine and Biology Society (EMBC),* 2016.
6. Janissen, R., Sahoo, P., Santos, C., Silva, A., Zuben, A., Souto, D., Costa, A., Celedon, Zanchin, P., N., Almeida, D., Oliveira, D., Kubota, L., Cesar, C., Souza, A., and Cotta, M., InP Nanowire Biosensor with Tailored Biofunctionalization: Ultrasensitive and Highly Selective Disease Biomarker Detection, *ACS Nano Lett.* **17**, 5938-5949 (2017).
7. Lei, Y. M., Xiao, M. M., Li, Y. T., Xu, L., Zhang, H., Zhang, Z. Y., and Zhang, G., Detection of heart failure-related biomarker in whole blood with graphene field effect transistor biosensor, *Biosens Bioelectron.* **91**, 1-7 (2017).
8. Kwon, J., Lee, Y., Lee, T., and Ahn, J., Aptamer-Based Field-Effect Transistor for Detection of Avian Influenza Virus in Chicken Serum, *ACS Anal. Chem.* **92**, 5524–5531 (2020).
9. Tzouvadaki, I., Stathopoulos, S., Abbey, T., Michalas, L., and Prodromakis, T., Monitoring PSA levels as chemical state-variables in metal-oxide memristors, *Sci. Rep.* **10**, 15281(1-6) (2020).
10. Kumar, P., Maikap, S., Ginnaram, S., Qiu, J., Jana, D., Chakrabarti, S., Samanta, S., Singh, K., Roy, A., Jana, S., Dutta, M., Chang, Y., Cheng, H., Mahapatra, R., Chiu, H., and Yang, J., Cross-Point Resistive Switching Memory and Urea Sensing by Using Annealed GdOx Film in IrOx/GdOx/W Structure for Biomedical Applications, *J. Electrochem. Soc.* **164**, B127-B135 (2017).
11. Knapic, D. et al. Anodic Niobium–Titanium Oxide Crossbar Memristor Arrays for pH Sensing in Liquids. *Phys. Status Solidi A.,* 221, 2300878 (2024).
12. Sinhaa, S., Pal, T., Kumara, D., Sharma, R., Kharbanda, D., Khanna, P. K., Mukhiya, R., Design, fabrication and characterization of TiN sensing film-based ISFET pH sensor, *Mater. Lett.* **304**, 130556 (2021).
13. Kim, H., Kim, J., Jeong, M., Lee, D., Kim, J., Lee, M., Kim, G., Kim, J., Lee, J., and Lee, J., Bioelectronic Sutures with Electrochemical pH-Sensing for Long-Term Monitoring of the Wound Healing Progress, *Adv. Funct. Mater*. **34**, 2402501 (2024).
14. Zhou, G. Full hardware implementation of neuromorphic visual system based on multimodal optoelectronic resistive memory arrays for versatile image processing. *Nat. Commun*. **14**, 8489 (2023).
15. Doremaele, E.R.W. et al. A retrainable neuromorphic biosensor for on-chip learning and classification. *Nat. Electron.* **6**, 765-770 (2023).
16. Liu, D. et al. A wearable in-sensor computing platform based on stretchable organic electrochemical transistors. *Nat. Electron.* **7**, 1176-1185 (2024).
17. Ji, J. et al. Single-transistor organic electrochemical neurons. *Nat. Commun.* **16**, 4334 (2025).
18. Holt, B.M., Stine, J.M., Beardslee, L.A., Ayansola, H., Jin, Y., Pasricha, P.J., and Ghodssi, R., An ingestible bioimpedance sensing device for wireless monitoring of epithelial barriers. *Microsyst Nanoeng.* **11**, 24 (2025).
19. Even, A., Minderhoud, R., Torfs, T., Leonardi, F., van Heusden, A., Sijabat, R., Firfilionis, D., Castro Miller, I. D., Rammouz, R., Teichmann, T., van Bergen, R., Vermeeren, G., Capuano, E., Armstrong, R., Mathwig, K., de Vries, S., Goris, A., Van Helleputte, N., Hooiveld, G., Van Hoof, C. Measurements of Redox Balance along the Gut Using a Miniaturized Ingestible Sensor. *Nat. Electron* (2025).
20. Sun, D., Zhu, X., Chen, S., Fang, H., Zhu, G., Lan, G., He, L., and Shi, Y. Uniformity, Linearity, and Symmetry Enhancement in TiOx/MoS2−xOx Based Analog RRAM via S-Vacancy Confined Nanofilament. *Nano Lett.* **24**, 16283-16292 (2024).
21. Lee, J., Lee, W.-C. & Cho, H.-J. Resistive Memory Device With 1T-1R and The Fabrication Method Of The Same,

Korean Patent 10-2820732-0000, (2025). Available at: https://www.kipris.or.kr/khome/search/searchResult.do
