# Supplementary material for: Demonstration of CMOS-compatible memristor-based electrochemical biosensor transducer with threshold-sensing functionality
Source: Nat Commun. 2025 Dec 3;16:10851. doi: 10.1038/s41467-025-66372-w (PMC12675689; doi:10.1038/s41467-025-66372-w)
Supplement: Supplementary file 2 — Description of Additional Supplementary Information [file 41467_2025_66372_MOESM2_ESM.pdf]

Description of Additional Supplementary Information

- Supplementary Video 1: Operation of the proposed memristor-based electrochemical biosensor transducer with threshold-sensing functionality towards pH monitoring

- Supplementary Video 2: Operation of the proposed memristor-based electrochemical biosensor transducer with threshold-sensing functionality towards glucose detection

- Supplementary Video 3: Operation of the proposed memristor-based electrochemical biosensor transducer with threshold-sensing functionality towards ascorbic acid detection
